# Corrigendum to “Expression and Gene Regulation Network of Metabolic Enzyme Phosphoglycerate Mutase Enzyme 1 in Breast Cancer Based on Data Mining”

**DOI:** 10.1155/2021/9764265

**Published:** 2021-07-10

**Authors:** Yongxuan Wang, Xifeng Xiong, Xing Hua, Wei Liu

**Affiliations:** ^1^Department of Pathology, Guangzhou Red Cross Hospital, Jinan University, Guangzhou, Guangdong 510220, China; ^2^Guangzhou Institute of Traumatic Surgery, Guangzhou Red Cross Hospital, Jinan University, Guangzhou, Guangdong 510220, China; ^3^Department of Breast Surgery, Guangzhou Red Cross Hospital, Jinan University, Guangzhou, Guangdong 510220, China

In the article titled “Expression and Gene Regulation Network of Metabolic Enzyme Phosphoglycerate Mutase Enzyme 1 in Breast Cancer Based on Data Mining” [1], Figure 5 included additional parts (b–y) which should have been omitted. The corrected figure is shown as follows.

## Figures and Tables

**Figure 1:**
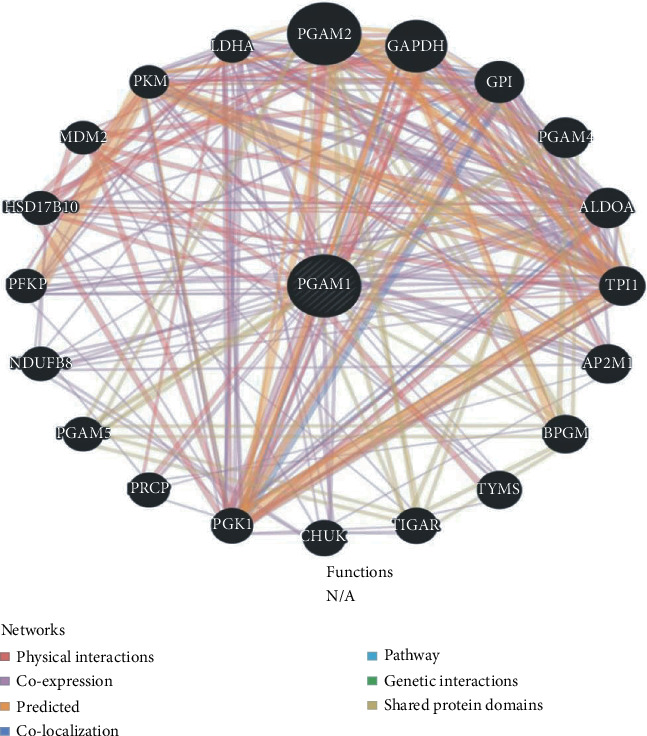
Protein-protein interaction (PPI) network and functional analysis of PGAM1. The different colors at the edge of the network indicate the applied bioinformatics methods: coexpression, website prediction, pathway, physical interaction, and colocalization.
